# Molecular Mechanisms of Intimal Hyperplasia in Saphenous Vein Grafts After Coronary Artery Bypass Grafting

**DOI:** 10.3390/cells15171520

**Published:** 2026-08-24

**Authors:** Dejan M. Lazovic, Dragan Cvetkovic, Milica Karadzic Kocica, Selena Nesic, Dragan Ivanisevic, Vojkan Aleksic, Mladen J. Kocica, Jovana Klac, Danko Grujic, Vladimir Jovicic, Stefan Juricic

**Affiliations:** 1 Clinic for Cardiac Surgery, University Clinical Center of Serbia, 11000 Belgrade, Serbiavaleksicc@gmail.com (V.A.);; 2 Faculty of Medicine, University of Belgrade, 11000 Belgrade, Serbia; 3 Center for Anesthesiology, Reanimatology and Intensive Care Medicine, University Clinical Center of Serbia, 11000 Belgrade, Serbia; 4 Clinic for Cardiology, University Clinical Center of Serbia, 11000 Belgrade, Serbia

**Keywords:** CABG, vein graft, intimal hyperplasia, vascular smooth muscle cells, inflammation, endothelial dysfunction

## Abstract

Coronary artery disease is a leading cause of morbidity and mortality in modern medicine. In contrast, surgical myocardial revascularization via coronary artery bypass grafting (CABG) remains the gold standard of treatment for complex multivessel disease. The great saphenous vein remains the most frequently used conduit due to its availability and technical simplicity, but its long-term patency is significantly inferior to that of arterial grafts. The primary pathological process responsible for vein graft failure is intimal hyperplasia, which represents a complex response of the vascular wall to surgical trauma, vein arterialization, inflammation, and hemodynamic stress. This process is characterized by endothelial dysfunction, inflammatory cell activation, proliferation and migration of vascular smooth muscle cells, and extracellular matrix remodeling. Underpinning these alterations are numerous molecular pathways, including NF-κB, MAPK, PI3K/Akt, TGF-β, and mTOR signaling, as well as substantial contributions from oxidative stress, cytokines, growth factors, and microRNAs. Contemporary research indicates that the phenotypic transformation of vascular smooth muscle cells constitutes the central event in the development of intimal hyperplasia. Understanding the cellular and molecular mechanisms underlying this disease’s onset enables the development of novel therapeutic strategies to preserve long-term graft patency. This review paper aims to provide a systematic overview of current knowledge regarding the molecular and cellular mechanisms of intimal hyperplasia development in vein grafts following CABG.

## 1. Introduction

Coronary artery disease represents one of the most critical healthcare challenges in the modern world. Despite significant advancements in percutaneous coronary intervention, coronary surgery continues to serve as the cornerstone therapeutic modality for patients with complex multivessel disease, diabetes, and left main coronary artery stenosis [1]. The great saphenous vein is used in most CABG procedures owing to its accessibility, length, and technical convenience. However, the long-term patency of vein grafts remains limited. It is established that approximately 10–15% of grafts thrombose within the first year, whereas up to 40–50% of vein grafts exhibit significant stenosis after 10 years [2].

In preparing this review paper, a systematic search of the literature on the pathophysiology of vein graft intimal hyperplasia following coronary artery bypass grafting (CABG) was conducted. The literature search was conducted using electronic databases including PubMed, Scopus, Web of Science, and Google Scholar. Articles published between 1990 and 2026 were analyzed, with particular focus on contemporary research from the last ten years. The keywords and combinations utilized included: ‘vein graft intimal hyperplasia’, ‘saphenous vein graft failure’, ‘vascular smooth muscle cells’, ‘endothelial dysfunction’, ‘vascular inflammation’, ‘molecular pathways’, ‘oxidative stress’, ‘microRNA’, ‘CABG’, ‘vein graft remodeling’, and ‘phenotypic switching’. The search was restricted to articles published in the English language and available in full text.

Inclusion criteria comprised original experimental and clinical studies, review articles, meta-analyses, and translational research evaluating the molecular, cellular, and inflammatory mechanisms of vein graft intimal hyperplasia development after CABG. Particular attention was dedicated to studies investigating the role of vascular smooth muscle cells, endothelial dysfunction, oxidative stress, cytokines, signaling pathways, and epigenetic mechanisms in disease development. Papers with insufficient methodological data, small sample sizes lacking clear statistical analysis, publications not directly related to vein graft pathophysiology, and non-peer-reviewed publications were excluded from the analysis. After removing duplicate records, approximately 320 publications were screened by title and abstract. Following full-text evaluation, the most relevant studies were selected based on scientific quality, clinical relevance, and their contribution to understanding the cellular and molecular mechanisms of vein graft remodeling. A total of 54 references were ultimately included in this review, comprising original research articles, systematic reviews, meta-analyses, and recent translational studies. Preference was given to high-quality publications, particularly those published within the last five years, to provide an up-to-date overview of current knowledge and emerging therapeutic strategies.

Following the initial screening of titles and abstracts, articles holding the highest scientific and clinical relevance to the scope of this literature review were thoroughly analyzed.

**Figure 1:**
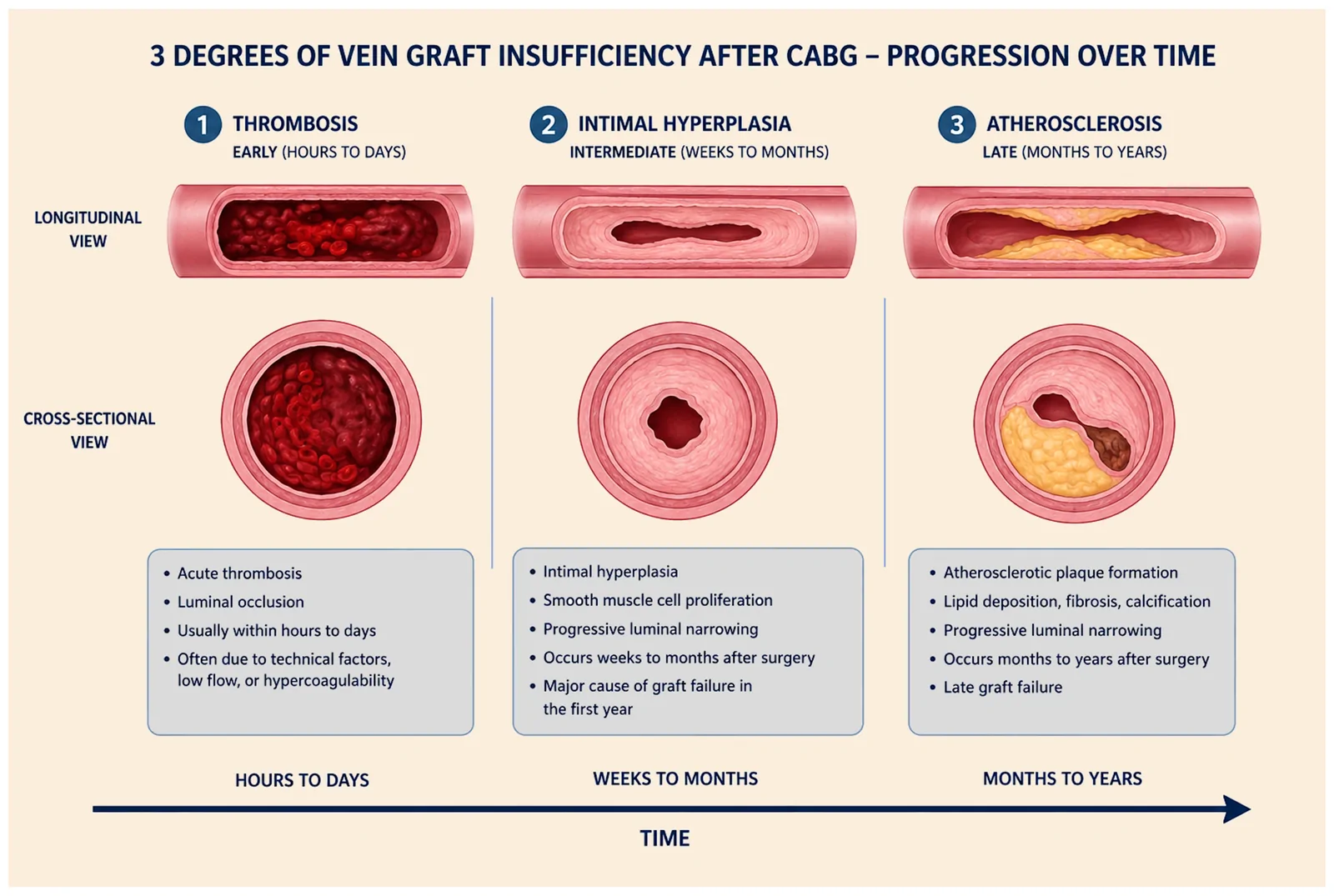
Progression of Saphenous Vein Graft Failure After coronary artery bypass graft (CABG). This schematic illustration depicts the temporal evolution of saphenous vein graft failure following coronary artery bypass grafting (CABG).

The pathophysiology of vein graft failure unfolds through three distinct phases: early thrombosis, intimal hyperplasia, and late graft atherosclerosis (Figure 1). Intimal hyperplasia serves as a critical transitional event linking early thrombosis to late atherosclerosis. The process arises as a response of the vein to its exposure to arterial pressure and altered shear stress forces following implantation into the arterial system [3]. This adaptive response involves endothelial activation, inflammatory infiltration, migration, and proliferation of vascular smooth muscle cells (VSMCs), as well as accelerated extracellular matrix synthesis [4,5].

Modern molecular biology has provided a deeper understanding of the signaling pathways involved in the development of intimal hyperplasia. Particular interest has centered on the NF-κB, MAPK, and TGF-β signaling pathways, which regulate inflammation, cellular proliferation, and vascular remodeling [6,7].

## 2. Histological and Functional Characteristics of the Vein Graft

**Figure 2:**
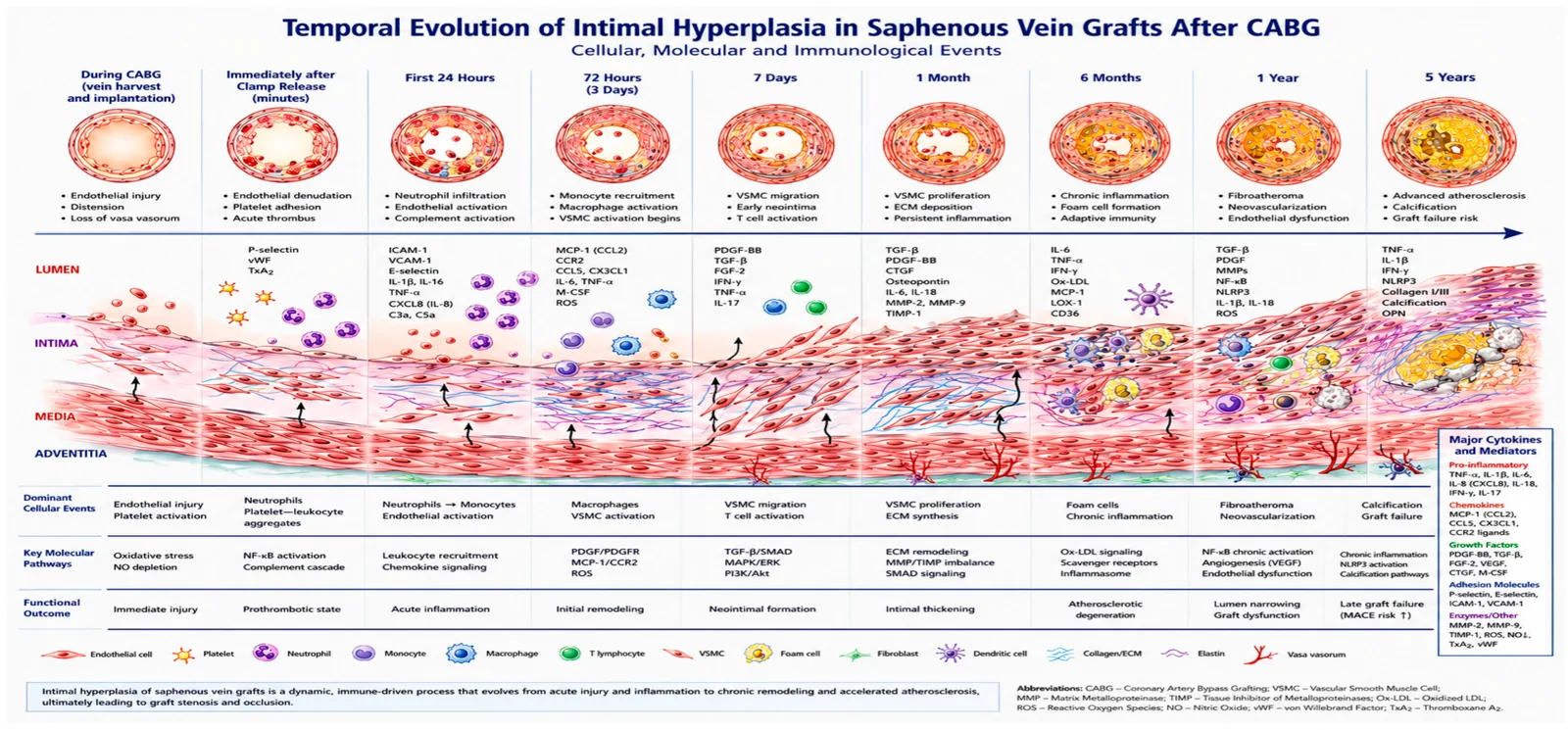
Temporal evolution of intimal hyperplasia in saphenous vein grafts after coronary artery bypass grafting (CABG): cellular, molecular, and immunological mechanisms. The figure illustrates the progression of intimal hyperplasia and accelerated vein graft disease following coronary artery bypass grafting (CABG), emphasizing the sequential cellular, molecular, and immunological events from the moment of graft implantation through late graft failure. The pathological process begins during graft harvesting and implantation, when the saphenous vein is exposed to mechanical trauma, ischemia–reperfusion injury, overdistension, and sudden arterial hemodynamic stress. These factors result in endothelial injury, loss of nitric oxide bioavailability, oxidative stress, and disruption of the vasa vasorum, thereby initiating an acute inflammatory and prothrombotic response.

The great saphenous vein is physiologically adapted to low-pressure, low-shear-stress conditions. Upon implantation into the arterial system, an abrupt shift in biomechanical conditions occurs. Veins are immediately subjected to high arterial pressure, pulsatile flow, and increased tangential stress. This sudden change activates mechanosensitive receptors and triggers a complex vascular remodeling process. The normal structure of the vein wall comprises a thinner media with fewer smooth muscle cells and a less developed internal elastic lamina compared to arteries. The venous endothelium exhibits an attenuated capacity for nitric oxide production and heightened susceptibility to oxidative stress. These precise anatomical and functional features contribute to its predisposition toward developing intimal hyperplasia [8,9]. The lining of our blood vessels, called the endothelium, plays a crucial role in keeping our vascular system healthy. But when we harvest and prepare veins for grafting, the endothelium is damaged by handling, stretching, and reduced blood flow. This damage reduces the production of important substances such as nitric oxide and prostacyclin, which help our blood vessels relax and prevent blood clots. At the same time, the damaged endothelium begins producing more adhesion molecules, such as VCAM-1, ICAM-1, and selectins, which attract white blood cells to the area. These white blood cells then stick to the blood vessel wall and move into the surrounding tissue, causing inflammation. The endothelium also releases proinflammatory cytokines such as TNF-α, IL-1β, and IL-6, which worsen inflammation (Figure 2). Immediately after clamp release, endothelial denudation promotes platelet adhesion and activation, leading to the subsequent release of thromboxane A2, P-selectin, and von Willebrand factor. Activated platelets interact with circulating leukocytes and stimulate recruitment of neutrophils through chemokine-mediated signaling pathways. Concurrently, complement activation and reactive oxygen species (ROS) generation amplify endothelial dysfunction and vascular inflammation.

Within the first 24 h, the inflammatory response intensifies with marked neutrophil infiltration and monocyte adhesion to the activated endothelium. Upregulation of adhesion molecules, including ICAM-1, VCAM-1, and E-selectin, facilitates leukocyte transmigration into the vessel wall. Activation of the NF-κB signaling pathway induces transcription of multiple proinflammatory cytokines, including TNF-α, IL-1β, IL-6, and IL-8, thereby propagating vascular inflammation and endothelial apoptosis [8].

By 72 h, macrophages become the predominant inflammatory cells within the graft wall. Simultaneously, vascular smooth muscle cells (VSMCs) transition from a contractile to a synthetic phenotype under the influence of platelet-derived growth factor (PDGF), transforming growth factor-β (TGF-β), fibroblast growth factor-2 (FGF-2), and vascular endothelial growth factor (VEGF). Matrix metalloproteinases (MMP-2 and MMP-9) degrade extracellular matrix components, facilitating migration of VSMCs from the media into the intimal layer. Adventitial fibroblast activation further contributes to vascular remodeling [9].

At approximately 7 days after implantation, early neointimal formation becomes evident. Proliferation and migration of VSMCs are accompanied by accumulation of macrophages and activation of T lymphocytes. Intracellular signaling pathways including MAPK/ERK and PI3K/Akt regulate cellular proliferation, survival, and extracellular matrix synthesis. Increased deposition of collagen, fibronectin, and proteoglycans progressively thickens the intimal layer [10].

One month after CABG, intimal hyperplasia becomes well established. Synthetic phenotype VSMCs dominate the neointima, while persistent macrophage-mediated inflammation maintains chronic cytokine production. Elevated levels of osteopontin, MCP-1, IL-18, and macrophage colony-stimulating factor (M-CSF) perpetuate leukocyte recruitment and extracellular matrix remodeling. An imbalance between matrix metalloproteinases and their tissue inhibitors of metalloproteinases (TIMPs) further promotes pathological vascular remodeling and luminal narrowing.

At six months, the pathological process gradually transitions from isolated intimal hyperplasia toward accelerated graft atherosclerosis. Chronic inflammatory infiltrates persist within the vessel wall, and foam cells accumulate as macrophages take up oxidized low-density lipoprotein (ox-LDL) via scavenger receptor-mediated pathways. Ongoing activation of inflammatory mediators such as IL-6 and TNF-α contributes to endothelial senescence and progressive vascular degeneration.

By one year, fibroatheromatous plaque formation becomes increasingly apparent. Chronic NF-κB activation, oxidative stress, and reduced endothelial nitric oxide synthase (eNOS) activity sustain endothelial dysfunction and inflammatory signaling. Neovascularization of the neointima may occur through VEGF-mediated angiogenic pathways, while persistent macrophage and T-cell activity promotes plaque progression and instability.

At five years, advanced vein graft disease is characterized by diffuse fibroatherosclerosis, calcification, chronic inflammation, and plaque instability. Activation of the NLRP3 inflammasome and sustained production of TNF-α, IL-1β, and IFN-γ maintain chronic immune dysregulation and oxidative injury. Extensive extracellular matrix remodeling, fibrosis, and calcification ultimately lead to severe graft stenosis, thrombosis, myocardial ischemia, and late graft failure [11].

Overall, the figure demonstrates that intimal hyperplasia in saphenous vein grafts is not a single, isolated event but rather a complex, temporally coordinated process involving endothelial dysfunction, innate and adaptive immune activation, vascular smooth muscle cell phenotypic transformation, extracellular matrix remodeling, and progressive atherosclerotic degeneration.

All these changes can trigger a response that leads to thickening of the blood vessel wall, known as intimal hyperplasia. A key player in this process is the NF-κB signaling pathway, which controls the expression of genes involved in inflammation and the production of cytokines and adhesion molecules. When this pathway is activated, it triggers a chain reaction that drives the inflammatory response and subsequent changes in the blood vessel wall [10,11].

## 3. Inflammation and Immune Response

The inflammatory response represents the principal driver of intimal hyperplasia development. Following endothelial injury, neutrophils, monocytes, and T-lymphocytes are recruited into the graft wall. Monocytes differentiate into macrophages, which secrete numerous proinflammatory mediators and growth factors. Macrophages release PDGF, TGF-β, FGF, and VEGF, which directly stimulate the proliferation and migration of vascular smooth muscle cells. Concurrently, the generation of reactive oxygen species (ROS) further amplifies inflammation and endothelial injury [12,13]. T-lymphocytes exert important regulatory functions through the production of interferon-γ and other cytokines that modulate macrophage and VSMC activity. Chronic inflammation sustains this proliferative response and drives lesion progression [14].

The central cellular event in the development of intimal hyperplasia is the phenotypic transformation of vascular smooth muscle cells from a contractile to a synthetic phenotype (Table 1). Immediately after graft reperfusion, endothelial injury, platelet activation, oxidative stress, and inflammatory cell recruitment initiate a complex cascade of vascular remodeling. During the first 24 h, neutrophil infiltration and activation of pro-inflammatory signaling pathways amplify endothelial dysfunction and promote leukocyte adhesion and transmigration [15].

A critical transition occurs approximately 72 h after graft implantation, when vascular smooth muscle cells (VSMCs) begin to undergo phenotypic switching from a contractile to a synthetic phenotype, representing one of the earliest and most important events in the development of intimal hyperplasia. Under the influence of growth factors such as platelet-derived growth factor (PDGF), transforming growth factor-β (TGF-β), fibroblast growth factor-2 (FGF-2), and vascular endothelial growth factor (VEGF), VSMCs lose their differentiated contractile characteristics and acquire enhanced proliferative, migratory, and extracellular matrix–producing capabilities. Concurrent activation of matrix metalloproteinases (particularly MMP-2 and MMP-9) facilitates degradation of the extracellular matrix and internal elastic structures, enabling VSMC migration from the medial layer toward the intima [16].

By the end of the first week, synthetic VSMCs become the dominant cellular component of the developing neointima. These cells actively proliferate and secrete extracellular matrix proteins, including collagen, elastin, fibronectin, and proteoglycans, leading to progressive neointimal formation. The process is further sustained by macrophage-derived cytokines and activation of signaling pathways such as MAPK/ERK and PI3K/Akt. At one month and beyond, persistent synthetic VSMC activity and chronic low-grade inflammation contribute to progressive luminal narrowing and structural graft remodeling [17]. Overall, the table highlights that although endothelial injury initiates the pathological process, the early phenotypic transformation of VSMCs, occurring as early as 72 h after CABG, is the pivotal biological event driving subsequent intimal hyperplasia and long-term vein graft failure. Understanding the molecular mechanisms that regulate this phenotypic switch may identify important therapeutic targets to improve long-term graft patency [18].

Under physiological conditions, VSMCs maintain vascular tone and express contractile proteins such as α-SMA, SM22α, and calponin. Under the influence of inflammatory cytokines, oxidative stress, and hemodynamic alterations, VSMCs shed their contractile phenotype and acquire proliferative and migratory features. The synthetic phenotype is characterized by accelerated proliferation, migration into the intima, and extensive extracellular matrix synthesis [15]. A crucial regulator of this process is PDGF-BB, which activates the MAPK and PI3K/Akt signaling pathways. TGF-β further stimulates the synthesis of collagen and fibronectin, while the mTOR signaling pathway regulates cellular growth and proliferation.

Phenotypic transformation of vascular smooth muscle cells (VSMCs) is currently recognized as the central cellular event in the initiation and progression of vein graft intimal hyperplasia after CABG [16,17]. Under physiological conditions, the VSMCs within the media of the venous wall reside in a so-called contractile phenotype, characterized by minimal proliferative activity and dominant expression of contractile proteins, including α-smooth muscle actin (α-SMA), smooth muscle myosin heavy chain (SM-MHC), calponin, and SM22α [17]. Their fundamental functions are to regulate vascular tone and maintain the structural integrity of the vessel wall.

Following implantation of the saphenous vein into the arterial system, an abrupt transformation in biomechanical and hemodynamic conditions occurs. Veins are exposed to high arterial pressure, elevated circumferential stress, and altered shear stress forces. Simultaneously, surgical manipulation, graft distension, and ischemia–reperfusion injury lead to endothelial breakdown and the release of inflammatory mediators [4,6]. These stimuli activate intracellular signaling cascades that induce a phenotypic switch of VSMCs from a contractile to a synthetic state.

The synthetic phenotype is characterized by increased proliferation, migration from the media to the intima, heightened extracellular matrix synthesis, and downregulated expression of contractile proteins [16]. These cells become metabolically hyperactive and acquire the capacity to produce type I and III collagens, fibronectin, proteoglycans, and matrix metalloproteinases. This results in progressive intimal thickening and vascular wall remodeling. VSMC migration represents a critical step in neointima development. For cells to migrate through the internal elastic lamina, matrix metalloproteinases—specifically MMP-2 and MMP-9—are activated to degrade the extracellular matrix, thereby permitting cellular translocation [18,19]. Following intimal migration, VSMCs undergo intense proliferation driven by growth factors and cytokines.

Platelet-derived growth factor (PDGF), particularly the PDGF-BB isoform, plays a uniquely pivotal role because it is released from activated platelets, macrophages, and endothelial cells [14]. Binding of PDGF to the PDGFR-β receptor triggers a series of signaling cascades that induce VSMC proliferation and migration. Beyond PDGF, substantial contributions are made by transforming growth factor-beta (TGF-β), fibroblast growth factor (FGF), epidermal growth factor (EGF), and insulin-like growth factor-1 (IGF-1). Contemporary studies demonstrate that VSMCs possess exceptional phenotypic plasticity [20]. Aside from the synthetic phenotype, certain subpopulations can exhibit macrophage-like, osteochondrogenic, or fibroblastic phenotypes, thereby further driving the progression of vascular remodeling and graft atherosclerosis [15].

## 4. Molecular Signaling Pathways in Intimal Hyperplasia

**NF-κB Signaling Pathway**

The nuclear factor-kappa B (NF-κB) signaling pathway is one of the central regulators of inflammation and vascular remodeling during vein graft adaptation following coronary artery bypass grafting (CABG). Following implantation of the saphenous vein into the arterial circulation, endothelial injury, oxidative stress, ischemia–reperfusion injury, and altered hemodynamic forces rapidly activate NF-κB signaling in endothelial cells, vascular smooth muscle cells (VSMCs), macrophages, and infiltrating immune cells [21]. Under physiological conditions, NF-κB remains inactive in the cytoplasm through its interaction with inhibitor of κB (IκB) proteins [22]. Various pathological stimuli, including reactive oxygen species (ROS), tumor necrosis factor-α (TNF-α), interleukin-1β (IL-1β), and Toll-like receptor (TLR) activation, induce phosphorylation and degradation of IκB by the IκB kinase (IKK) complex, allowing NF-κB to translocate into the nucleus. Once activated, NF-κB promotes the transcription of numerous pro-inflammatory genes, including TNF-α, IL-6, IL-1β, monocyte chemoattractant protein-1 (MCP-1), vascular cell adhesion molecule-1 (VCAM-1), and intercellular adhesion molecule-1 (ICAM-1), thereby amplifying leukocyte recruitment and sustaining chronic vascular inflammation [11,17]. Persistent activation of NF-κB also stimulates VSMC proliferation and migration while promoting phenotypic switching from the contractile to the synthetic phenotype, a hallmark of intimal hyperplasia [23]. Furthermore, NF-κB enhances extracellular matrix remodeling by increasing the expression of matrix metalloproteinases (MMP-2 and MMP-9) and reducing the activity of tissue inhibitors of metalloproteinases (TIMPs), thereby facilitating structural changes within the vein graft wall. Cross-talk between NF-κB and other signaling pathways, including PI3K/Akt, MAPK, TGF-β/SMAD, Notch, Hippo/YAP-TAZ, and the NLRP3 inflammasome, further amplifies inflammatory and proliferative responses [24]. Recent studies have demonstrated that mitochondrial dysfunction and excessive ROS generation establish a positive feedback loop that maintains sustained NF-κB activation during chronic vascular remodeling. In addition, several microRNAs, including miR-21, miR-146a, and miR-155, regulate NF-κB signaling, highlighting the close interaction between inflammatory and epigenetic mechanisms during vein graft disease [25]. Single-cell transcriptomic analyses have further revealed that NF-κB activity varies among distinct VSMC, endothelial, and macrophage subpopulations, suggesting that cell-specific modulation of this pathway may improve therapeutic precision [26]. Experimental inhibition of NF-κB signaling using pharmacological inhibitors, antioxidant therapies, or gene-silencing approaches has consistently reduced inflammatory cytokine production, VSMC proliferation, and neointimal formation in preclinical models [27,28]. Collectively, these findings identify NF-κB as a master regulator integrating inflammatory, oxidative, and mechanical signals during vein graft remodeling and support its potential as a promising therapeutic target for improving long-term vein graft patency after CABG [29].

**MAPK Signaling Pathway**

The mitogen-activated protein kinase (MAPK) signaling pathway represents one of the principal intracellular signaling cascades regulating vascular remodeling during vein graft adaptation after coronary artery bypass grafting (CABG). Mechanical stretch, increased arterial pressure, ischemia–reperfusion injury, oxidative stress, inflammatory cytokines, and growth factors rapidly activate MAPK signaling within endothelial cells, vascular smooth muscle cells (VSMCs), fibroblasts, and infiltrating immune cells [30]. The MAPK family comprises three major signaling branches, extracellular signal-regulated kinase (ERK1/2), c-Jun N-terminal kinase (JNK), and p38 MAPK, each regulating distinct yet interconnected biological processes [31]. Activation of the ERK1/2 pathway is primarily stimulated by platelet-derived growth factor (PDGF), epidermal growth factor (EGF), fibroblast growth factor (FGF), and vascular endothelial growth factor (VEGF), promoting VSMC proliferation, migration, and survival. ERK1/2 signaling also enhances cell-cycle progression through the upregulation of cyclin D1 and cyclin-dependent kinases, thereby accelerating neointimal formation. In contrast, the stress-responsive kinases JNK and p38 MAPK are predominantly activated by reactive oxygen species (ROS), inflammatory mediators, hypoxia, and mechanical injury [32,33]. Persistent activation of JNK stimulates the transcription factor activator protein-1 (AP-1), which induces the expression of pro-inflammatory cytokines, matrix metalloproteinases, and apoptosis-related genes. Similarly, p38 MAPK promotes inflammatory responses by regulating the synthesis of tumor necrosis factor-α (TNF-α), interleukin-1β (IL-1β), interleukin-6 (IL-6), and monocyte chemoattractant protein-1 (MCP-1), thereby facilitating leukocyte recruitment into the vessel wall [34,35]. MAPK signaling also plays a crucial role in vascular smooth muscle cell phenotypic switching from the contractile to the synthetic phenotype, characterized by increased proliferation, migration, extracellular matrix production, and reduced expression of contractile proteins such as α-smooth muscle actin (α-SMA), smooth muscle myosin heavy chain (MYH11), and calponin. Furthermore, MAPK activation stimulates the production of matrix metalloproteinases (MMP-2 and MMP-9), which degrade extracellular matrix components and facilitate vascular remodeling and neointimal expansion [36]. Crosstalk between MAPK signaling and the PI3K/Akt, NF-κB, TGF-β/SMAD, Notch, Hippo/YAP-TAZ, and Wnt/β-catenin pathways amplifies inflammatory and proliferative responses during vein graft disease [37]. Oxidative stress further enhances MAPK activation through NADPH oxidase-derived ROS, establishing a positive feedback loop that perpetuates chronic vascular inflammation. Recent evidence also indicates that mitochondrial dysfunction contributes to sustained MAPK activation by increasing intracellular oxidative stress and altering cellular energy metabolism. Emerging studies have demonstrated that several non-coding RNAs, including miR-143/145, miR-21, miR-221/222, and multiple long non-coding RNAs, modulate MAPK signaling during vascular remodeling, highlighting an important interaction between epigenetic regulation and intracellular signaling. Single-cell RNA sequencing has further revealed heterogeneous activation of MAPK signaling among distinct VSMC and macrophage subpopulations, suggesting that only specific cellular subsets may drive pathological remodeling. Experimental inhibition of ERK1/2, JNK, or p38 MAPK significantly reduces VSMC proliferation, inflammatory cytokine production, extracellular matrix deposition, and neointimal formation in animal models of vein graft disease [38]. Pharmacological inhibitors targeting individual MAPK components have shown encouraging results in preclinical studies, although their systemic use remains limited by potential adverse effects related to the physiological roles of MAPK signaling in multiple organs. Consequently, local drug delivery systems, nanoparticle-based therapies, and hydrogel-mediated vascular applications are being investigated to selectively inhibit MAPK activity within vein grafts while minimizing systemic toxicity. Future therapeutic strategies may combine MAPK inhibition with modulation of epigenetic regulators, RNA-based therapeutics, and gene-editing technologies to achieve more effective suppression of pathological vascular remodeling [39,40]. Collectively, the MAPK signaling pathway functions as a central integrator of mechanical, inflammatory, oxidative, and growth factor-mediated stimuli and represents one of the most promising molecular targets for preventing intimal hyperplasia and improving long-term vein graft patency following CABG [41,42].

**PI3K/Akt/mTOR Signaling Pathway**

**Figure 3:**
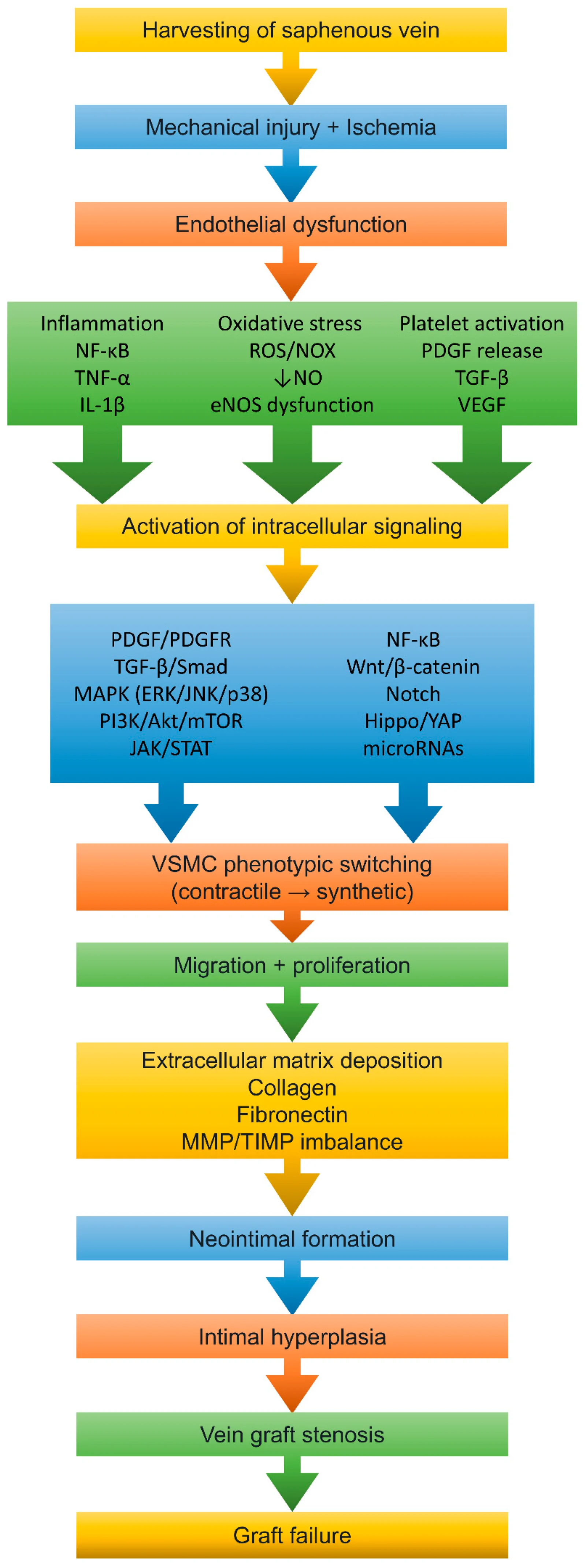
Molecular mechanisms underlying saphenous vein graft intimal hyperplasia following coronary artery bypass grafting (CABG). Mechanical injury and ischemia during saphenous vein harvesting initiate endothelial dysfunction, leading to inflammation, oxidative stress, and platelet activation. These processes activate multiple intracellular signaling pathways, including PDGF/PDGFR, TGF-β/Smad, MAPK, PI3K/Akt/mTOR, JAK/STAT, NF-κB, Wnt/β-catenin, Notch, Hippo/YAP, and microRNAs, which promote vascular smooth muscle cell (VSMC) phenotypic switching, migration, proliferation, extracellular matrix deposition, and ultimately neointimal formation.

The phosphatidylinositol 3-kinase (PI3K)/Akt/mammalian target of rapamycin (mTOR) signaling pathway is a fundamental intracellular regulatory network that governs cell proliferation, survival, metabolism, migration, protein synthesis, and angiogenesis during vascular remodeling [19]. Following coronary artery bypass grafting (CABG), exposure of the saphenous vein to the arterial environment induces rapid activation of the PI3K/Akt/mTOR pathway in endothelial cells, vascular smooth muscle cells (VSMCs), fibroblasts, and infiltrating inflammatory cells (Figure 3). Mechanical stretch, oxidative stress, ischemia–reperfusion injury, platelet-derived growth factor (PDGF), vascular endothelial growth factor (VEGF), insulin-like growth factor-1 (IGF-1), and inflammatory cytokines serve as major upstream activators of PI3K signaling. Upon activation, PI3K catalyzes the conversion of phosphatidylinositol-4,5-bisphosphate (PIP2) into phosphatidylinositol-3,4,5-trisphosphate (PIP3), facilitating the recruitment and phosphorylation of Akt at the plasma membrane [43]. Activated Akt subsequently phosphorylates numerous downstream substrates involved in cell survival, metabolism, proliferation, migration, and protein synthesis. One of its most important downstream targets is the mechanistic target of rapamycin (mTOR), which exists in two functionally distinct complexes, mTORC1 and mTORC2. Activation of mTORC1 stimulates protein synthesis through phosphorylation of ribosomal protein S6 kinase (S6K1) and eukaryotic translation initiation factor 4E-binding protein-1 (4E-BP1), thereby promoting VSMC growth and extracellular matrix production. In contrast, mTORC2 contributes to cytoskeletal organization, cell migration, and complete activation of Akt, further enhancing vascular remodeling [44,45].

Persistent activation of the PI3K/Akt/mTOR pathway promotes the phenotypic transition of VSMCs from a quiescent contractile phenotype to a highly proliferative synthetic phenotype characterized by increased migration, extracellular matrix secretion, and reduced expression of contractile proteins, including α-smooth muscle actin (α-SMA), smooth muscle myosin heavy chain (MYH11), calponin, and SM22α. Simultaneously, PI3K/Akt signaling enhances endothelial cell survival by suppressing apoptosis and stimulating endothelial nitric oxide synthase (eNOS) activity through Akt-dependent phosphorylation, thereby increasing nitric oxide bioavailability during the early phases of vascular repair. However, prolonged or dysregulated activation of this pathway contributes to pathological neointimal thickening by sustaining VSMC proliferation and chronic inflammatory responses. PI3K/Akt/mTOR signaling also regulates macrophage polarization, favoring the accumulation of pro-inflammatory macrophage subsets that release cytokines and growth factors promoting vascular remodeling. Furthermore, activation of this pathway increases the expression of matrix metalloproteinases (MMP-2 and MMP-9), facilitating extracellular matrix degradation, cellular migration, and structural remodeling of the vein graft wall [46,47].

Extensive cross-talk exists between PI3K/Akt/mTOR and several other signaling pathways implicated in intimal hyperplasia, including MAPK/ERK, NF-κB, TGF-β/SMAD, Hippo/YAP-TAZ, Wnt/β-catenin, and Notch signaling. Reactive oxygen species generated by NADPH oxidases further amplify PI3K/Akt activation, establishing a positive feedback loop that perpetuates oxidative stress and inflammation. Emerging evidence also demonstrates that multiple non-coding RNAs, including miR-21, miR-126, miR-143/145, miR-221/222, MALAT1, and H19, directly regulate PI3K/Akt/mTOR signaling, highlighting the intimate relationship between epigenetic regulation and intracellular signaling during vein graft remodeling. Single-cell RNA sequencing studies have further demonstrated heterogeneous activation of PI3K/Akt signaling among distinct VSMC and endothelial cell subpopulations, suggesting that only specific cellular subsets contribute disproportionately to pathological neointimal development [48].

Pharmacological inhibition of mTOR with rapamycin (sirolimus) and its analogues has consistently reduced VSMC proliferation and neointimal formation in numerous experimental models and has formed the biological basis for drug-eluting vascular devices [49,50]. More recently, nanoparticle-based drug delivery systems and hydrogel-mediated local administration of mTOR inhibitors have demonstrated enhanced efficacy while minimizing systemic adverse effects. Current experimental strategies also include selective PI3K isoform inhibitors, Akt inhibitors, RNA-based therapeutics, and CRISPR/Cas9-mediated modulation of pathway components. These targeted interventions aim to suppress pathological vascular remodeling while preserving physiological endothelial repair. Collectively, the PI3K/Akt/mTOR signaling pathway functions as a central molecular hub integrating growth factor signaling, inflammatory activation, oxidative stress, and mechanical stimuli during vein graft adaptation, making it one of the most promising therapeutic targets for preventing intimal hyperplasia and improving long-term vein graft patency after CABG [51].

**TGF-β/Smad Signaling Pathway**

The transforming growth factor-beta (TGF-β)/SMAD signaling pathway is one of the most important regulators of vascular remodeling, extracellular matrix homeostasis, and vascular smooth muscle cell (VSMC) phenotypic modulation during vein graft adaptation following coronary artery bypass grafting (CABG) [52]. Following implantation of the saphenous vein into the arterial circulation, endothelial injury, ischemia–reperfusion damage, oxidative stress, and inflammatory cytokines stimulate the local production and activation of TGF-β, primarily TGF-β1, by endothelial cells, VSMCs, macrophages, fibroblasts, and activated platelets. TGF-β binds to the type II transforming growth factor-beta receptor (TGFβRII), which subsequently recruits and phosphorylates the type I receptor (TGFβRI), initiating intracellular signal transduction. Activated TGFβRI phosphorylates the receptor-regulated SMAD proteins, SMAD2 and SMAD3, which subsequently associate with the common mediator SMAD4 and translocate into the nucleus. Within the nucleus, SMAD complexes regulate the transcription of numerous genes involved in cell proliferation, differentiation, extracellular matrix synthesis, fibrosis, inflammation, and vascular remodeling. Physiologically, TGF-β signaling contributes to vascular homeostasis by maintaining endothelial integrity and regulating extracellular matrix turnover. However, sustained activation following vein graft implantation promotes pathological remodeling characterized by excessive collagen deposition, fibronectin accumulation, and increased synthesis of proteoglycans, ultimately leading to progressive intimal thickening [53,54].

TGF-β signaling is a major driver of VSMC phenotypic switching, promoting the transition from the differentiated contractile phenotype to the synthetic phenotype that exhibits enhanced proliferative, migratory, and matrix-producing capacities. Activated SMAD3 induces the expression of extracellular matrix proteins, including collagen types I and III, fibronectin, connective tissue growth factor (CTGF), and plasminogen activator inhibitor-1 (PAI-1), thereby contributing to fibrosis and neointimal expansion. Simultaneously, TGF-β suppresses matrix degradation by reducing the activity of matrix metalloproteinases while increasing tissue inhibitors of metalloproteinases (TIMPs), favoring extracellular matrix accumulation within the vein graft wall. Beyond its effects on VSMCs, TGF-β also modulates macrophage polarization, fibroblast activation, and endothelial cell function, thereby orchestrating multiple cellular processes involved in chronic vascular remodeling. Recent studies have further demonstrated that TGF-β induces endothelial-to-mesenchymal transition (EndMT), during which endothelial cells acquire mesenchymal characteristics, lose endothelial markers such as CD31 and VE-cadherin, and contribute directly to the pool of matrix-producing cells within the neointima [55].

Accumulating evidence indicates extensive cross-talk between TGF-β/SMAD signaling and multiple intracellular pathways implicated in intimal hyperplasia, including PI3K/Akt/mTOR, MAPK/ERK, NF-κB, Notch, Hippo/YAP-TAZ, and Wnt/β-catenin signaling. These interactions amplify inflammatory responses, extracellular matrix remodeling, and VSMC proliferation. Mechanical forces generated by arterial pressure and cyclic stretch further enhance TGF-β activation through integrin-mediated release of latent TGF-β from the extracellular matrix, establishing a mechanotransduction-dependent positive feedback loop. Oxidative stress also potentiates TGF-β signaling by increasing reactive oxygen species production through NADPH oxidases, while activated TGF-β further stimulates oxidative stress, creating a self-perpetuating cycle of vascular injury. Emerging evidence has demonstrated that several non-coding RNAs, including miR-21, miR-29, miR-145, MALAT1, H19, and multiple circular RNAs, directly regulate TGF-β/SMAD signaling, highlighting the important interaction between epigenetic regulation and vascular remodeling. Single-cell transcriptomic analyses have identified distinct fibroblast, endothelial, and VSMC subpopulations characterized by enhanced TGF-β signaling activity, suggesting that cellular responses to TGF-β are highly heterogeneous within the vein graft wall [56].

Experimental inhibition of TGF-β signaling using neutralizing antibodies, receptor kinase inhibitors, antisense oligonucleotides, or selective SMAD3 inhibitors has consistently reduced extracellular matrix deposition, VSMC proliferation, fibrosis, and neointimal formation in experimental models of vascular injury. More recently, localized drug delivery systems, nanoparticle-based therapeutics, and RNA-targeting strategies have been developed to selectively modulate TGF-β signaling while minimizing systemic adverse effects associated with global inhibition of this pleiotropic cytokine. Future therapeutic approaches will likely combine TGF-β pathway modulation with epigenetic therapies, RNA-based therapeutics, and precision drug delivery systems to achieve more effective prevention of vein graft failure. Overall, the TGF-β/SMAD signaling pathway represents a central molecular regulator integrating inflammation, fibrosis, mechanotransduction, and extracellular matrix remodeling, making it one of the most attractive therapeutic targets for preventing intimal hyperplasia and improving long-term vein graft patency following CABG [48,57].

**Oxidative Stress Signaling Pathways**

Oxidative stress is a fundamental molecular mechanism driving vein graft remodeling and represents one of the earliest pathological events following coronary artery bypass grafting (CABG) [58]. Following implantation of the saphenous vein into the arterial circulation, the abrupt exposure to high-pressure pulsatile flow, increased oxygen tension, and altered shear stress results in excessive generation of reactive oxygen species (ROS) within the vessel wall. Although physiological levels of ROS function as important intracellular signaling molecules regulating vascular homeostasis, excessive ROS production disrupts redox balance and promotes endothelial dysfunction, vascular smooth muscle cell (VSMC) phenotypic switching, chronic inflammation, extracellular matrix remodeling, and ultimately intimal hyperplasia. Major enzymatic sources of ROS within the vein graft include nicotinamide adenine dinucleotide phosphate (NADPH) oxidases, mitochondrial electron transport chain dysfunction, xanthine oxidase, uncoupled endothelial nitric oxide synthase (eNOS), cyclooxygenases, and lipoxygenases [59].

Among these, the NADPH oxidase (NOX) family is considered the predominant source of pathological ROS during vascular remodeling. NOX1 and NOX2 are markedly upregulated following vascular injury and primarily stimulate VSMC proliferation, inflammatory activation, and leukocyte recruitment, whereas NOX4 appears to exert more complex context-dependent functions by regulating cellular differentiation and redox homeostasis. Increased ROS generated by NOX enzymes activate multiple intracellular signaling pathways, including NF-κB, MAPK/ERK, PI3K/Akt/mTOR, TGF-β/SMAD, Hippo/YAP-TAZ, and Wnt/β-catenin signaling, thereby amplifying inflammatory and proliferative responses throughout the vein graft wall [60].

Mitochondria constitute another major source of oxidative stress during vein graft adaptation. Ischemia–reperfusion injury occurring during graft harvesting and implantation disrupts mitochondrial respiration, resulting in excessive production of mitochondrial ROS (mtROS). Mitochondrial oxidative stress impairs ATP synthesis, alters calcium homeostasis, induces mitochondrial DNA damage, and activates apoptotic signaling pathways. Persistent mitochondrial dysfunction also establishes a positive feedback loop whereby ROS further damage mitochondrial proteins and respiratory chain complexes, perpetuating chronic oxidative injury. Recent studies have demonstrated that impaired mitophagy contributes to the accumulation of dysfunctional mitochondria, thereby sustaining oxidative stress during vascular remodeling [61].

Endothelial dysfunction represents one of the earliest consequences of excessive ROS generation. Oxidative stress reduces nitric oxide (NO) bioavailability through direct chemical interaction between NO and superoxide anions, leading to formation of highly reactive peroxynitrite (ONOO^−^). Peroxynitrite induces protein nitration, lipid peroxidation, DNA damage, and endothelial apoptosis while simultaneously oxidizing tetrahydrobiopterin (BH4), an essential cofactor required for normal endothelial nitric oxide synthase (eNOS) activity. Loss of BH4 results in eNOS uncoupling, whereby eNOS produces superoxide instead of nitric oxide, thereby further amplifying oxidative stress and endothelial dysfunction [62].

Oxidative stress profoundly influences vascular smooth muscle cell biology. Elevated ROS concentrations stimulate VSMC proliferation and migration while promoting phenotypic switching from the contractile phenotype toward the synthetic phenotype. This transition is accompanied by decreased expression of contractile proteins including α-smooth muscle actin (α-SMA), SM22α, calponin, and smooth muscle myosin heavy chain (MYH11), together with increased production of collagen, fibronectin, proteoglycans, and matrix metalloproteinases (MMP-2 and MMP-9). ROS also activate redox-sensitive transcription factors including NF-κB, activator protein-1 (AP-1), hypoxia-inducible factor-1α (HIF-1α), and nuclear factor erythroid 2-related factor 2 (Nrf2), thereby coordinating inflammatory and antioxidant responses [63].

Inflammatory cells further amplify oxidative injury within the vein graft. Activated macrophages and neutrophils release large quantities of superoxide, hydrogen peroxide, and hypochlorous acid through activation of NOX2 and myeloperoxidase. Simultaneously, inflammatory cytokines such as tumor necrosis factor-α (TNF-α), interleukin-1β (IL-1β), and interferon-γ further stimulate ROS production within endothelial cells and VSMCs, establishing a self-perpetuating inflammatory cycle [42].

Oxidative stress also contributes to extracellular matrix remodeling by increasing matrix metalloproteinase activity while suppressing tissue inhibitors of metalloproteinases (TIMPs). These alterations facilitate degradation of the vascular extracellular matrix, promote VSMC migration into the intima, and accelerate neointimal expansion. Furthermore, ROS stimulate fibroblast activation and collagen synthesis through TGF-β-dependent signaling, contributing to fibrosis and progressive vein graft stiffening [64].

Recent evidence indicates extensive interaction between oxidative stress and epigenetic regulation. Reactive oxygen species directly modify DNA methylation patterns, histone acetylation, and non-coding RNA expression, thereby altering transcriptional programs involved in vascular remodeling. Several microRNAs, including miR-21, miR-126, miR-143/145, miR-146a, and miR-155, respond to oxidative stress and subsequently regulate inflammatory signaling, endothelial repair, and VSMC phenotype. Long non-coding RNAs such as MALAT1 and H19 also participate in redox-sensitive signaling networks that modulate vascular remodeling [65].

Single-cell transcriptomic studies have recently demonstrated substantial heterogeneity in oxidative stress responses among endothelial cells, VSMCs, macrophages, fibroblasts, and perivascular immune cells. Certain VSMC subpopulations exhibit markedly increased expression of oxidative stress-related genes, including NOX1, NOX2, CYBA, and multiple antioxidant enzymes, suggesting that only specific cellular populations predominantly drive pathological remodeling. Integration of single-cell transcriptomics with spatial transcriptomics has further revealed regional differences in oxidative stress within the vein graft wall, providing new insight into disease progression [66].

Therapeutic strategies targeting oxidative stress have shown encouraging experimental results. Pharmacological inhibition of NADPH oxidases, enhancement of Nrf2-dependent antioxidant responses, mitochondrial-targeted antioxidants such as MitoQ and SkQ1, restoration of tetrahydrobiopterin levels to recouple eNOS, and activation of endogenous antioxidant enzymes including superoxide dismutase (SOD), catalase, glutathione peroxidase, and heme oxygenase-1 (HO-1) have all demonstrated protective effects in experimental vascular injury models. More recently, nanoparticle-based drug delivery systems, RNA therapeutics targeting oxidative stress-related genes, and CRISPR/Cas9-mediated gene editing have emerged as promising approaches for selectively reducing oxidative injury within vein grafts while preserving physiological redox signaling [67].

Collectively, oxidative stress functions as a central integrator of endothelial dysfunction, inflammation, mitochondrial injury, extracellular matrix remodeling, and VSMC phenotypic switching during vein graft adaptation. Its extensive interactions with inflammatory, metabolic, and epigenetic pathways make oxidative stress one of the most important molecular mechanisms underlying intimal hyperplasia and an attractive therapeutic target for improving long-term vein graft patency after CABG [26,30].

**MicroRNA and Epigenetic Regulation**

MicroRNAs (miRNAs) are small endogenous non-coding RNA molecules approximately 20–24 nucleotides in length that regulate gene expression at the post-transcriptional level by binding to complementary sequences within target messenger RNAs (mRNAs). Through translational repression or mRNA degradation, miRNAs regulate virtually every aspect of vascular biology, including endothelial homeostasis, vascular smooth muscle cell (VSMC) differentiation, inflammation, oxidative stress, extracellular matrix remodeling, apoptosis, and angiogenesis. Increasing evidence indicates that dysregulation of miRNA expression represents one of the earliest molecular events initiating vein graft remodeling following coronary artery bypass grafting (CABG). Altered hemodynamic forces, ischemia–reperfusion injury, platelet activation, oxidative stress, and inflammatory cytokines rapidly modify the expression profile of numerous vascular miRNAs, thereby orchestrating the transition from physiological adaptation toward pathological intimal hyperplasia [35,40,44,45,46].

Among the best-characterized vascular microRNAs is the miR-143/145 cluster, which serves as a master regulator of VSMC differentiation. Under physiological conditions, miR-143 and miR-145 maintain the contractile phenotype by promoting the expression of myocardin, smooth muscle α-actin (ACTA2), smooth muscle myosin heavy chain (MYH11), calponin, and SM22α while simultaneously suppressing genes associated with proliferation and migration. Following vein graft implantation, expression of the miR-143/145 cluster decreases markedly, resulting in phenotypic switching of VSMCs toward the synthetic phenotype characterized by enhanced proliferation, migration, extracellular matrix production, and inflammatory activation. Experimental restoration of miR-145 expression has consistently reduced neointimal formation and preserved vascular integrity in animal models [57,65,68].

Conversely, miR-21 is consistently upregulated after vascular injury and functions as one of the principal mediators of pathological vascular remodeling. Increased miR-21 expression promotes VSMC proliferation and survival by suppressing phosphatase and tensin homolog (PTEN), thereby enhancing PI3K/Akt/mTOR signaling. In addition, miR-21 stimulates transforming growth factor-beta (TGF-β)/SMAD signaling and extracellular matrix synthesis while inhibiting apoptosis of vascular smooth muscle cells. Elevated miR-21 also contributes to macrophage activation and chronic inflammation, establishing a positive feedback loop that accelerates intimal hyperplasia [64,69].

Another important regulatory molecule is miR-126, which is predominantly expressed in endothelial cells. MiR-126 maintains endothelial integrity by promoting angiogenesis, endothelial repair, nitric oxide bioavailability, and vascular homeostasis while suppressing leukocyte adhesion through inhibition of VCAM-1 expression. Reduced miR-126 levels after endothelial injury impair endothelial regeneration and facilitate inflammatory cell infiltration into the vein graft wall. Consequently, therapeutic delivery of miR-126 has emerged as a promising strategy for accelerating endothelial recovery after CABG [70].

Members of the miR-221/222 family play equally important roles in vascular remodeling. These microRNAs stimulate VSMC proliferation by suppressing cyclin-dependent kinase inhibitors p27^Kip1^ and p57^Kip2^ while simultaneously reducing the expression of contractile proteins. Increased expression of miR-221/222 has been associated with accelerated neointimal formation in both arterial and venous models of vascular injury. Similarly, miR-146a functions as a regulator of inflammatory signaling by modulating NF-κB activity and Toll-like receptor signaling, whereas miR-155 amplifies macrophage-mediated inflammatory responses and oxidative stress during vascular remodeling.

Recent investigations have demonstrated that microRNAs rarely function independently but instead participate in highly interconnected regulatory networks involving long non-coding RNAs (lncRNAs), circular RNAs (circRNAs), transcription factors, chromatin-modifying enzymes, and intracellular signaling pathways. According to the competing endogenous RNA (ceRNA) hypothesis, lncRNAs and circRNAs function as molecular sponges that bind microRNAs and prevent them from suppressing their downstream target genes. Consequently, dysregulation of a single non-coding RNA may influence the expression of hundreds of genes simultaneously, providing an explanation for the remarkable phenotypic plasticity observed during vascular remodeling [71].

Epigenetic regulation extends far beyond non-coding RNAs and also includes DNA methylation, histone modifications, chromatin remodeling, and higher-order chromatin organization. DNA methylation, mediated by DNA methyltransferases (DNMT1, DNMT3A, and DNMT3B), generally suppresses transcription by adding methyl groups to cytosine residues within CpG islands. During vein graft remodeling, abnormal DNA methylation patterns alter the expression of genes controlling inflammation, oxidative stress, extracellular matrix synthesis, endothelial function, and VSMC differentiation. Hypermethylation of protective genes and hypomethylation of pro-inflammatory genes contribute to persistent vascular remodeling and chronic inflammation [72].

Histone modifications represent another major layer of epigenetic regulation. Histone acetyltransferases (HATs) promote chromatin relaxation and transcriptional activation, whereas histone deacetylases (HDACs) generally induce chromatin condensation and transcriptional repression. Likewise, histone methyltransferases and demethylases dynamically regulate gene accessibility depending on the specific lysine residues modified. Several studies have demonstrated increased HDAC activity within neointimal lesions, contributing to VSMC proliferation, inflammatory activation, and extracellular matrix accumulation. Pharmacological HDAC inhibitors have therefore shown promising anti-proliferative and anti-inflammatory effects in experimental vascular injury models [73].

Epigenetic regulation is closely integrated with multiple intracellular signaling pathways, including PI3K/Akt/mTOR, MAPK/ERK, NF-κB, TGF-β/SMAD, Hippo/YAP-TAZ, Notch, and Wnt/β-catenin signaling. Mechanical stress, oxidative injury, disturbed shear stress, and inflammatory mediators modify epigenetic enzyme activity, which subsequently regulates transcriptional programs responsible for vascular remodeling. Conversely, activated signaling pathways also modify chromatin accessibility and non-coding RNA expression, establishing complex bidirectional regulatory networks [42].

The recent introduction of single-cell transcriptomics, single-cell epigenomics (scATAC-seq), and spatial transcriptomics has revolutionized the understanding of epigenetic regulation in vascular biology. These technologies have revealed previously unrecognized heterogeneity among VSMCs, endothelial cells, fibroblasts, macrophages, and immune cells within the vein graft wall, demonstrating that epigenetic landscapes differ substantially between individual cellular subpopulations. Integration of single-cell multiomics with artificial intelligence is expected to identify novel therapeutic targets and predictive biomarkers for vein graft failure [65].

Collectively, accumulating evidence indicates that microRNAs and epigenetic regulation function as master regulators coordinating inflammatory signaling, cellular plasticity, extracellular matrix remodeling, endothelial repair, and vascular smooth muscle cell phenotype during vein graft adaptation. Their reversible nature makes epigenetic mechanisms particularly attractive therapeutic targets. Future strategies combining RNA-based therapeutics, selective epigenetic modulators, targeted nanoparticle delivery systems, and precision medicine approaches may fundamentally transform the prevention of intimal hyperplasia and significantly improve long-term vein graft patency following coronary artery bypass grafting [74].

## 5. Therapeutic Strategies

Given that intimal hyperplasia arises from the combined impact of surgical trauma, endothelial dysfunction, inflammation, hemodynamic stress, and vascular smooth muscle cell proliferation, prevention must target all phases of the CABG procedure—from vein harvesting and intraoperative graft handling to postoperative pharmacological and metabolic control (Table 2). During vein harvesting and graft preparation, techniques such as no-touch harvesting, controlled low-pressure distension, and optimized graft preservation solutions primarily aim to prevent endothelial injury and reduce oxidative stress. Preservation of endothelial nitric oxide bioavailability and vasa vasorum integrity is crucial for limiting early inflammatory activation and phenotypic switching of VSMCs.

Intraoperative preventive measures emphasize optimizing graft geometry and hemodynamics, including appropriate anastomotic configuration, avoidance of graft kinking or competitive flow, and use of external support devices to reduce circumferential wall stress and mechanotransduction-mediated cellular activation. These interventions are directed toward maintaining laminar flow patterns and preventing low-shear-stress-induced endothelial dysfunction [64].

Postoperative pharmacological strategies primarily target thromboinflammatory and proliferative pathways. Antiplatelet agents reduce platelet-derived growth factor (PDGF) release and thrombus formation. At the same time, statins exert pleiotropic anti-inflammatory and endothelial-protective effects through inhibition of NF-κB signaling and improvement of endothelial nitric oxide synthase (eNOS) activity. Additional therapies, including ACE inhibitors, angiotensin receptor blockers, antioxidants, and mTOR inhibitors, modulate oxidative stress, transforming growth factor beta (TGF-β) signaling, and VSMC proliferation.

The table also includes emerging therapeutic approaches such as microRNA modulation, gene therapy, epigenetic regulation, and local drug-delivery systems, which represent promising future strategies for targeted inhibition of neointimal formation and long-term preservation of graft patency. Overall, the summarized mechanisms demonstrate that preventing vein graft intimal hyperplasia requires a multifactorial, biologically integrated approach that preserves endothelial homeostasis, suppresses inflammatory signaling, and modulates vascular remodeling [20].

The ‘no-touch’ vein-harvesting technique preserves perivascular tissue and minimizes endothelial breakdown, thereby significantly improving long-term graft patency. Avoiding excessive manual distension and executing gentle tissue manipulation attenuates the acute inflammatory response. Aspirin and statins form the baseline of pharmacological prevention against graft failure. Statins exhibit powerful pleiotropic benefits, including suppression of vascular inflammation and inhibition of VSMC proliferation [20]. mTOR inhibitors such as sirolimus have demonstrated profound efficacy in reducing intimal hyperplasia. Antioxidants and NF-κB inhibitors likewise represent viable therapeutic targets. Current cutting-edge research is heavily focused on gene therapies directed against proliferative signaling axes, while microRNA-based therapeutics stand out as highly promising avenues for future clinical implementation [19].

Despite significant advances in surgical techniques and pharmacotherapy, intimal hyperplasia remains the principal cause of long-term saphenous vein graft failure after coronary artery bypass grafting (CABG). Current therapeutic strategies have evolved from conventional antithrombotic treatment toward targeted interventions aimed at modulating vascular smooth muscle cell (VSMC) phenotypic switching, endothelial dysfunction, inflammation, and extracellular matrix remodeling [64,69,75].

Optimization of surgical vein harvesting techniques remains the first preventive step. The no-touch harvesting technique preserves endothelial integrity, vasa vasorum, and perivascular adipose tissue, thereby reducing inflammatory activation and subsequent neointimal formation [36,44]. In parallel, meticulous graft preservation and avoidance of excessive intraluminal distension minimize endothelial injury immediately after graft implantation.

Pharmacological therapy continues to represent the cornerstone of secondary prevention. Statins exert pleiotropic effects beyond lipid lowering by improving endothelial nitric oxide bioavailability, suppressing oxidative stress, inhibiting NF-κB activation, and attenuating VSMC proliferation [64,76]. Likewise, renin–angiotensin system inhibitors reduce transforming growth factor-β (TGF-β)-mediated fibrosis and vascular remodeling, whereas prolonged dual antiplatelet therapy may reduce thrombo-inflammatory responses during the early postoperative period [64].

Recent studies have focused on molecular therapies directly targeting VSMC phenotypic plasticity. Inhibition of the PI3K/Akt/mTOR pathway using sirolimus or everolimus effectively suppresses VSMC proliferation and extracellular matrix deposition. Novel perivascular drug-delivery systems, including sirolimus-loaded silk microneedle wraps, have demonstrated sustained local drug release with significant inhibition of neointimal hyperplasia while minimizing systemic adverse effects [77].

Gene-based therapeutic approaches have emerged as promising strategies for preventing vein graft failure. Increasing evidence indicates that microRNAs are master regulators of VSMC phenotype. Restoration of contractile microRNAs, including miR-143 and miR-145, or inhibition of pathological microRNAs such as miR-21 and miR-18a-5p, suppresses VSMC proliferation, migration, and synthetic transformation, thereby limiting neointimal formation [16,69,78,79]. Local viral and non-viral gene delivery systems are currently being investigated to achieve sustained modulation of these molecular targets.

Advances in epigenetic therapy have further expanded potential treatment options. Pharmacological modulation of histone acetylation, DNA methylation, and chromatin remodeling has shown encouraging experimental results by stabilizing the contractile VSMC phenotype and reducing vascular inflammation [64,80,81].

Cell-based and immune-targeted therapies represent another emerging field. Experimental studies suggest that selective elimination of pathological synthetic VSMCs using engineered immune cells, including CAR-T-cell-based strategies, may significantly attenuate intimal hyperplasia while preserving physiological vascular repair mechanisms [16,79].

The rapid development of single-cell transcriptomics, spatial transcriptomics, artificial intelligence, and multi-omics technologies is expected to facilitate patient-specific therapeutic strategies by identifying molecular signatures associated with vein graft remodeling and treatment responsiveness. These technologies may ultimately enable precision medicine approaches capable of preventing vein graft failure before irreversible structural remodeling occurs [16,64,82].

Overall, future therapeutic strategies are expected to shift from nonspecific pharmacological inhibition toward personalized molecular interventions targeting VSMC phenotypic switching, endothelial regeneration, inflammatory signaling, and epigenetic regulation. Such multimodal approaches hold considerable promise for improving long-term vein graft patency and clinical outcomes following CABG.

## 6. Conclusions

Vein graft intimal hyperplasia after CABG is a highly intricate, multifactorial biological cascade encompassing endothelial dysfunction, robust inflammation, vascular smooth muscle cell proliferation, and extracellular matrix remodeling. The pivotal biological event is the phenotypic switching of VSMCs driven by a synergistic combination of inflammatory cytokines, local oxidative stress, and dramatic alterations in arterial hemodynamics. Contemporary molecular research has identified a myriad of regulatory signaling networks involved in this disease, including the NF-κB, MAPK, TGF-β, and PI3K/Akt/mTOR pathways. Fully elucidating these pathways opens critical avenues for targeted molecular therapies to prevent vein graft disease. Although refinement of surgical techniques and modern medical therapies have optimized outcomes after CABG, vein graft failure persists as a serious clinical obstacle. Future investigations must prioritize personalized therapeutic approaches and the rapid translation of molecular discoveries into bedside clinical practice.

## Figures and Tables

**Table 1:** Temporal evolution of cellular and molecular events leading to saphenous vein graft intimal hyperplasia after coronary artery bypass grafting (CABG). This table summarizes the sequential biological processes occurring in saphenous vein grafts following implantation into the arterial circulation.

Time After CABG	Dominant Cellular Events	Molecular/Cytokine Profile	Clinical/Vascular Changes	Clinical/Histopathological Consequences
**Minutes–Hours**	Endothelial cell injury; platelet adhesion and activation; neutrophil recruitment; complement activation	ROS, TNF-α, IL-1β, IL-6, thromboxane A2, P-selectin, vWF, MCP-1/CCL2	Mechanical stretch injury from arterialization; endothelial denudation; nitric oxide depletion	Acute endothelial dysfunction; initiation of thrombogenic and inflammatory cascade
**First 24 Hours**	Massive neutrophil infiltration; monocyte adhesion; platelet–leukocyte interactions	ICAM-1, VCAM-1, E-selectin, NF-κB activation, CXCL8/IL-8, C3a/C5a	Enhanced leukocyte transmigration; oxidative stress-mediated endothelial apoptosis	Early inflammatory activation and prothrombotic state
**72 Hours (3 Days)**	Transition from neutrophil to macrophage predominance; activation of VSMCs and adventitial fibroblasts	PDGF, TGF-β, FGF-2, VEGF, MMP-2, MMP-9, continued IL-6 signaling	Beginning of VSMC migration from media to intima; extracellular matrix degradation	Initiation of initial thickening and vascular remodeling
**7 Days**	Proliferation of VSMCs; macrophage accumulation; activation of T lymphocytes	TGF-β, PDGF-BB, IFN-γ, TNF-α, collagen synthesis pathways, MAPK/ERK and PI3K/Akt	Formation of early neointima; extracellular matrix deposition	Histologically evident intimal hyperplasia begins
**1 Month**	Dominance of synthetic phenotype VSMCs; persistent macrophage-mediated inflammation	Osteopontin, MCP-1, IL-18, M-CSF, persistent MMP/TIMP imbalance	Progressive neointimal expansion; reduced lumen diameter; incomplete endothelial regeneration	Established intimal hyperplasia with progressive graft remodeling
**6 Months**	Chronic inflammatory infiltrate; foam cell formation; adaptive immune activation	IL-6, CRP-associated pathways, oxidized LDL signaling, scavenger receptor activation	Accelerated atherosclerotic transformation; lipid accumulation; endothelial senescence	Intermediate graft stenosis and transition toward graft atherosclerosis
**1 Year**	Mature neointimal VSMCs; macrophage-derived foam cells; chronic T-cell activity	NF-κB, TGF-β, LOX-1, oxidative stress signaling, reduced eNOS activity	Fibroatheromatous plaque development; calcification pathways initiation	Clinically significant graft narrowing and reduced long-term patency
**5 Years**	Chronic immune dysregulation; advanced macrophage and foam-cell burden	TNF-α, IL-1β, IFN-γ, NLRP3 inflammasome activation, chronic oxidative injury	Advanced graft atherosclerosis; plaque instability; fibrosis and calcification	Late vein graft failure, thrombosis, myocardial ischemia, or recurrent angina

**Table 2:** Principles and mechanisms of prevention of saphenous vein graft intimal hyperplasia after coronary artery bypass grafting (CABG). The table summarizes the major preventive strategies aimed at preventing intimal hyperplasia in saphenous vein grafts following CABG, organized by the perioperative and postoperative phases of graft management. The presented approaches include surgical, pharmacological, molecular, hemodynamic, and experimental strategies aimed at preserving endothelial integrity, reducing inflammatory activation, inhibiting vascular smooth muscle cell (VSMC) proliferation and migration, and minimizing extracellular matrix remodeling.

Time After CABG Events	Dominant Cellular Events	Molecular/Cytokine Profile	Clinical/Vascular Changes	Clinical/Histopathological Consequences
**Minutes–Hours**	Endothelial cell injury; platelet adhesion and activation; neutrophil recruitment; complement activation	ROS, TNF-α, IL-1β, IL-6, thromboxane A2, P-selectin, vWF, MCP-1/CCL2	Mechanical stretch injury from arterialization; endothelial denudation; nitric oxide depletion	Acute endothelial dysfunction; initiation of thrombogenic and inflammatory cascade
**First 24 Hours**	Massive neutrophil infiltration; monocyte adhesion; platelet–leukocyte interactions	ICAM-1, VCAM-1, E-selectin, NF-κB activation, CXCL8/IL-8, C3a/C5a	Enhanced leukocyte transmigration; oxidative stress-mediated endothelial apoptosis	Early inflammatory activation and prothrombotic state
**72 Hours (3 Days)**	Transition from neutrophil to macrophage predominance; activation of VSMCs and adventitial fibroblasts	PDGF, TGF-β, FGF-2, VEGF, MMP-2, MMP-9, continued IL-6 signaling	Beginning of VSMC migration from media to intima; extracellular matrix degradation	Initiation of intimal thickening and vascular remodeling
**7 Days**	Proliferation of VSMCs; macrophage accumulation; activation of T lymphocytes	TGF-β, PDGF-BB, IFN-γ, TNF-α, collagen synthesis pathways, MAPK/ERK and PI3K/Akt	Formation of early neointima; extracellular matrix deposition	Histologically evident intimal hyperplasia begins
**1 Month**	Dominance of synthetic phenotype VSMCs; persistent macrophage-mediated inflammation	Osteopontin, MCP-1, IL-18, M-CSF, persistent MMP/TIMP imbalance	Progressive neointimal expansion; reduced lumen diameter; incomplete endothelial regeneration	Established intimal hyperplasia with progressive graft remodeling
**6 Months**	Chronic inflammatory infiltrate; foam cell formation; adaptive immune activation	IL-6, CRP-associated pathways, oxidized LDL signaling, scavenger receptor activation	Accelerated atherosclerotic transformation; lipid accumulation; endothelial senescence	Intermediate graft stenosis and transition toward graft atherosclerosis
**1 Year**	Mature neointimal VSMCs; macrophage-derived foam cells; chronic T-cell activity	NF-κB, TGF-β, LOX-1, oxidative stress signaling, reduced eNOS activity	Fibroatheromatous plaque development; calcification pathways initiation	Clinically significant graft narrowing and reduced long-term patency
**5 Years**	Chronic immune dysregulation; advanced macrophage and foam-cell burden	TNF-α, IL-1β, IFN-γ, NLRP3 inflammasome activation, chronic oxidative injury	Advanced graft atherosclerosis; plaque instability; fibrosis and calcification	Late vein graft failure, thrombosis, myocardial ischemia, or recurrent angina

## Data Availability

The authors confirm that the data supporting the findings of this study are available within the article.
